# Genetic variation in Southern USA rice genotypes for seedling salinity tolerance

**DOI:** 10.3389/fpls.2015.00374

**Published:** 2015-05-27

**Authors:** Teresa B. De Leon, Steven Linscombe, Glenn Gregorio, Prasanta K. Subudhi

**Affiliations:** ^1^School of Plant, Environmental, and Soil Sciences, Louisiana State University Agricultural CenterBaton Rouge, LA, USA; ^2^Rice Research Station, Louisiana State University Agricultural CenterRayne, LA, USA; ^3^Plant Breeding, Genetics, and Biotechnology Division, International Rice Research InstituteLos Baños, Philippines

**Keywords:** cluster analysis, multivariate analysis, *Oryza sativa*, physiological traits, Na^+^/K^+^ ratio, discriminant analysis, salinity tolerance, salt injury

## Abstract

The success of a rice breeding program in developing salt tolerant varieties depends on genetic variation and the salt stress response of adapted and donor rice germplasm. In this study, we used a combination of morphological and physiological traits in multivariate analyses to elucidate the phenotypic and genetic variation in salinity tolerance of 30 Southern USA rice genotypes, along with 19 donor genotypes with varying degree of tolerance. Significant genotypic variation and correlations were found among the salt injury score (SIS), ion leakage, chlorophyll reduction, shoot length reduction, shoot K^+^ concentration, and shoot Na^+^/K^+^ ratio. Using these parameters, the combined methods of cluster analysis and discriminant analysis validated the salinity response of known genotypes and classified most of the USA varieties into sensitive groups, except for three and seven varieties placed in the tolerant and moderately tolerant groups, respectively. Discriminant function and MANOVA delineated the differences in tolerance and suggested no differences between sensitive and highly sensitive (HS) groups. DNA profiling using simple sequence repeat markers showed narrow genetic diversity among USA genotypes. However, the overall genetic clustering was mostly due to subspecies and grain type differentiation and not by varietal grouping based on salinity tolerance. Among the donor genotypes, Nona Bokra, Pokkali, and its derived breeding lines remained the donors of choice for improving salinity tolerance during the seedling stage. However, due to undesirable agronomic attributes and photosensitivity of these donors, alternative genotypes such as TCCP266, Geumgangbyeo, and R609 are recommended as useful and novel sources of salinity tolerance for USA rice breeding programs.

## Introduction

Soil salinity is a worldwide problem in both irrigated and non-irrigated crop production, especially in coastal areas. The estimated cost of irrigation-induced salinity alone is about USD11billion yr^−1^ (Thomas and Morini, 2005). Excessive salt reduces growth, and induces leaf damage, necrosis, and eventual death of the crop.

Rice plants are generally susceptible to salinity. Seedlings die at salt level of 10 dSm^−1^ (Munns et al., 2006), and yield loss can be as high as 90% at 3.5 dSm^−1^ salt stress during the reproductive stage (Asch et al., 2000). Salinity tolerance is a complex trait, and phenotypic responses of plants to salinity stress are highly affected by the environment (Gregorio and Senadhira, 1993; Koyama et al., 2001; Flowers, 2004). Several highly tolerant (HT), traditional genotypes provide opportunities to improve salinity tolerance of rice through breeding. Collaborations of the International Rice Research Institute (IRRI) with researchers in India, Bangladesh, and the Philippines have led these breeding programs for salinity tolerance to considerable success (Gregorio et al., 2002).

In the United States, Louisiana is the third largest producer of rice (USDA National Statistics Service, 2013). However, its proximity to the Gulf of Mexico makes it vulnerable to salinity stress. During the hurricane season, salt water intrusion normally occurs in coastal areas. Moreover, if reduced rainfall follows the year of salt water flooding, fresh water gets contaminated with brackish water and recovery of affected areas becomes difficult (Leonards, 2012). After hurricanes Katrina, Rita, Gustav, and Ike, soil salinity increased in coastal areas of Louisiana. Soil salinity sampled from 2005 to 2008 ranged from 286 to 4329 parts per million (ppm) (Breitenbeck et al., 2007; Saichuk and Gauthier, 2011; Viator et al., 2011), while water salinity rose to as high as 7000 ppm between 2001 and 2003 (Branch, 2004).

The USA is a major exporter of rice to Latin America and Asia. Although Louisiana has considerable success for breeding high yielding rice varieties, continuous breeding is necessary to meet the demand of the world's increasing population in conjunction with changing climate, environment, and pests. However, successful targeted trait improvement depends on the availability of donor genotypes, efficient screening methods, and a thorough understanding of the genetics and physiology of salinity tolerance (Negrão et al., 2011).

Despite the establishment of a screening procedure for salinity tolerance by IRRI (Gregorio et al., 1997), consistency and reproducibility of results between laboratories worldwide remain a challenge due to the lack of uniform growth environments. Several studies have been published on the screening method (Yeo et al., 1990; Aslam et al., 1993; Asch et al., 2000), but only a few were in large scale (Yeo et al., 1990; Kanawapee et al., 2012). Although salinity tolerance is polygenic, most studies still treat salinity tolerance as a single trait and commonly use visual scoring (Gregorio et al., 1997) or the Na^+^/ K^+^ ratio for classification. Yeo et al. (1990) suggested pyramiding of favorable morphological and physiological traits to increase salinity tolerance. Therefore, a statistical model combining morphological and physiological traits would be more appropriate. Previously, cluster analysis using agronomic and physiological parameters has been employed in genotypic classification for salinity tolerance (Zeng et al., 2002). However, cross-validation of the clustering method was not employed to evaluate the accuracy of the classification. In addition, attempts to define the differences among levels of tolerance are not well established due to the complexity of tolerance and limited genotypic screening techniques (Platten et al., 2013). To address these concerns, we classified 49 rice genotypes for salinity tolerance based on the linear combination of morphological and physiological traits using the combined power of clustering and discriminant analyses. We employed MANOVA and canonical discriminant functions to define the differences in salinity tolerance. Lastly, we genotyped the 49 rice varieties to identify ideal tolerant genotypes suited for breeding programs in the Southern USA. To our knowledge, this is the first time these Southern USA rice varieties were evaluated for salinity tolerance and genetic relatedness.

## Materials and methods

### Plant materials

Forty-nine rice genotypes were screened for salinity tolerance at the seedling stage (Table 1 and Supplementary Table S1). Thirty varieties were grown in the Southern USA, and 14 genotypes were acquired from IRRI, including the sensitive check IR29 and the HT check Pokkali. The other five genotypes were acquired from the Germplasm Resources Information Network (GRIN).

**Table 1:** **Mean trait values of rice genotypes in salinity characterization**.

**Genotype**	**SIS**	**Ion leakage (uScm^−1^)**		**Ion_leak**	**Chlorophyll content (SPAD unit)**		**Chl_R**	**Root**		**RtL_R**	**Shoot**		**ShL_R**	**Rt_Na**	**Sh_Na**	**Rt_K**	**Sh_K**	**Rt_Na/K**	**Sh_Na/K**
		**Ro**	**Rt**	**(%)**	**Ctr**	**Sal**	**(%)**	**Ctr**	**Sal**	**(%)**	**Ctr**	**Sal**	**(%)**	**(mmol/kg)**	**(mmol/kg)**	**(mmol/kg)**	**(mmol/kg)**	**(Ratio)**	
Bengal	7.1	0.06	0.74	72	34	17	52	14	7	48	53	30	44	1444	2408	118	688	11	4.6
Caffey	6.2^*^	0.05	0.53	50	31	19	39	14	8	43	49	27	44	1737	2676	123	831	14	3.8
Catahoula	7.4	0.06	0.52	49	32	14	56	13	8	38	48	24	49	1721	2897	104	822	16	3.9
Cheniere	6.2^*^	0.06	0.71	69	31	23	27	12	8	38	46	23	51	1384	2582	103	785	12	3.5
Cheriviruppu	4.4^***^	0.04	0.36	33^*^	32	22	31	16	8	51	65	40	38	1576	2596	124	1120	13	2.3
CL 152	6.6	0.10	0.60	55	32	12	62	14	7	49	49	23	53^+^	1402	2846	116	897	25	3.2
CL111	7.5	0.04	0.47	44	35	15	56	15	8	49	53	25	53^+^	1144	2478	128	770	8	3.6
CL131	7.0	0.04	0.53	51	35	17	50	15	8	43	47	25	48	1710	2735	242	792	12	3.6
CL142	8.1	0.05	0.71	69	33	16	51	15	8	45	45	26	43	1863	2643	111	706	17	3.9
CL151	8.1	0.07	0.53	49	32	11	67	16	8	46	46	24	48	1892	2844	112	725	17	4.8
CL161	8.0	0.08	0.52	48	31	14	56	14	6	56	49	22	55^++^	1536	2614	130	738	12	3.7
CL162	6.0^*^	0.03	0.35	33^*^	34	20	42	15	7	52	48	24	49	1422	2702	123	894	11	3.1
CL181	7.6	0.07	0.46	42	34	16	53	13	8	41	39	21	46	1322	2714	108	716	11	4.3
CL261	8.7	0.13	0.47	40	32	6	80	15	9	38	51	28	45	1526	3158	147	840	10	3.8
Cocodrie	7.2	0.08	0.62	59	34	7	78	15	7	51	53	25	53^+^	1472	2534	116	860	11	3.0
CSR II (IRGC 83240)	3.8^***^	0.07	0.46	42	35	34	4^***^	14	9	37	41	23	43	1686	2517	150	861	12	3.0
Cypress	5.1^***^	0.04	0.43	40	32	21	36	15	7	53	51	26	48	1530	2493	130	731	12	3.7
Damodar (IRGC 17038)	5.0^***^	0.08	0.43	38	32	21	35	16	8	49	49	28	42	1702	2007	122	1333	15	2.1^*^
FL378	3.8^***^	0.04	0.33	30^*^	36	25	32	16	9	45	45	27	40	1623	3671	139	1336	12	2.8
FL478	3.0^***^	0.04	0.36	33^*^	33	27	18^***^	14	8	45	50	30	40	1299	2608	95	974	14	2.7
Getu (IRGC 17041)	3.9^***^	0.08	0.46	41	33	19	42	16	9	45	54	28	49	1806	3153	101	1032	18	3.0
Geumgangbyeo	3.9^***^	0.08	0.56	52	34	25	26	15	8	47	37	22	40	1545	2367	141	912	11	2.6
Hasawi (IRGC 16817)	4.0^***^	0.07	0.33	28^**^	30	21	31	15	9	41	62	41	34	1747	3203	141	1158	13	2.7
IR 1702-74-3-2	5.7^***^	0.06	0.50	51	32	18	42	15	9	42	44	24	45	1937	2958	151	1031	13	2.9
IR 2706-11-2	6.8	0.06	0.56	40	35	2	94	16	10	41	44	24	46	1572	3494	130	1103	12	3.2
IR 944-102-2-3-2	4.2^***^	0.11	0.46	47	33	27	19^***^	12	6	46	35	18	47	1246	2640	134	837	8	3.2
IR29	7.7	0.08	0.55	52	35	17	52	15	8	49	41	24	42	1751	3226	140	821	13	4.0
IRRI 147	6.1^**^	0.04	0.37	34	33	19	43	15	7	52	43	27	37	1532	2423	131	1002	12	2.4
Jazzman	6.9	0.07	0.54	51	31	15	52	12	6	51	52	22	58^+++^	1637	3362	112	921	15	3.9
Jazzman-2	7.5	0.04	0.45	42	32	14	56	13	7	46	47	24	50	1368	2158	113	614	11	3.5
Jes	7.2	0.04	0.48	46	30	16	46	12	7	44	36	21	42	1363	2702	106	629	12	4.7
Jupiter	6.2^*^	0.06	0.49	45	35	17	51	12	7	38	44	20	55^++^	1426	3297	154	916	8	3.7
Ketumbar (IRGC 13516)	5.8^**^	0.05	0.31	27^**^	31	19	39	18	12	34	49	29	42	1734	3574	156	1156	11	3.1
LA 0702085	8.6	0.04	0.65	63	32	4	89	13	6	52	51	24	52^+^	1894	2808	115	867	17	3.5
LA 0802140	7.6	0.05	0.52	50	32	12	64	15	8	45	50	23	54^+^	1471	2655	110	781	11	4.1
LAH 10	4.4^***^	0.04	0.58	56	35	23	33	14	8	47	48	26	46	1391	2269	135	794	10	2.9
Mermentau	7.1	0.09	0.58	54	34	7	80	14	8	44	53	27	49	1638	2776	140	745	11	4.1
Neptune	6.3^*^	0.05	0.51	48	34	21	37	13	8	40	50	26	47	1503	2935	119	765	12	4.5
Nipponbare	5.8^***^	0.08	0.56	53	35	24	32	15	7	52	41	22	46	1665	3241	138	909	12	3.6
Nona Bokra(IRGC 01231)	4.0^***^	0.04	0.37	34	32	19	40	16	9	42	70	44	37	1809	2832	129	1059	14	2.7
Pokkali (IRGC 108921)	2.9^***^	0.04	0.33	30^**^	33	21	35	16	7	54	70	46	34	1585	2702	109	995	15	2.7
PSBRC50 (IRGC 99706)	4.9^***^	0.06	0.41	38	36	24	32	14	8	42	43	23	45	2067	2702	171	1129	13	2.4
R609 (MG)	4.4^***^	0.07	0.61	58	34	24	31	12	6	48	45	24	47	1151	2673	108	774	10	3.7
Rex	7.0	0.05	0.52	49	33	17	48	17	8	51	49	25	49	1696	2570	110	791	15	3.4
Roy J	6.2^*^	0.06	0.75	73	35	19	47	13	7	44	46	24	48	1423	2949	116	863	10	3.7
Taggert	5.9^**^	0.05	0.53	51	34	14	60	13	8	40	45	25	44	1710	3235	111	761	16	5.1
TCCP-266-1-38-13-1-3	3.0^***^	0.05	0.41	38	33	25	24^*^	15	8	48	55	31	43	1566	2199	183	966	9	2.3
Templeton	6.0^*^	0.05	0.45	41	31	19	39	14	9	37	50	27	47	1657	2549	120	786	14	3.8
Wells	7.9	0.08	0.61	57	33	14	58	15	8	46	50	25	50	1762	2961	127	878	14	3.7
Genotypic effect (Pr > F)	<0.0001			<0.0001			<0.0001			0.993			<0.0001	0.847	0.086	0.376	0.049	0.262	0.016

*SIS, salt injury score; Ion_leak, index of injury by ion leakage; Ro, ion leakage in control treatment; Rt, ion leakage in saline treatment; Ctr, control; Sal, saline treatment; Chl_R, % Chlorophyll reduction; ShL_R, % shoot length reduction; RtL_R, % root length reduction; Rt_Na, Sodium concentration in root, Rt_Na/K, Na/K ratio in root; Sh_Na, Shoot sodium concentration (mmol/kg); Sh_K, shoot potassium concentration (mmol/kg); Sh_Na/K, Na/K ratio in shoot*.

* *Significantly different to IR29 at the 0.05 probability level*.

** *Significantly different to IR29 at the 0.01 probability level*.

*** *Significantly different to IR29 at the 0.001 probability level*.

+ *Significantly different to Pokkali at the 0.05 probability level*.

++ *Significantly different to Pokkali at the 0.01 probability level*.

+++ *Significantly different to Pokkali at the 0.001 probability level*.

### Screening for salinity tolerance at seedling stage

Unimbibed seeds of the 49 rice genotypes were incubated at 50°C for 5 days to break any residual seed dormancy. The IRRI standard evaluation technique (Gregorio et al., 1997) for salinity tolerance was followed with some modifications. Ten seeds from each genotype were pre-germinated in a paper towel for 2 days at 35°C and then transferred into a styrofoam trays suspended on a basin containing tap water. After 3 days, the seedlings were allowed to grow for 2 weeks in a hydroponic nutrient solution containing 1 g/l of Jack's Professional fertilizer 20-20-20 (J.R. Peters, Inc.) supplemented with 300 mg/l ferrous sulfate. NaCl was added to the nutrient solution at 12 dSm^−1^ with the pH maintained between 5.0 and 5.1. Control plants were grown at the same time in nutrient solutions without NaCl. All experiments were conducted in a greenhouse with temperatures set between 25 and 29°C.

The entire experiment was conducted in a randomized block design and was replicated three times. Ten seedlings were grown, but only five plants of uniform growth per genotype for every replication were considered for data collection. The mean value of the trait for five seedlings per genotype was considered one replicate.

#### Ion leakage

Early responses of rice genotypes to salinity stress were investigated by measuring the concentration of the ions that leaked from the leaf tissue using a conductivity meter (VWR Traceable). After 2 days in saline solution, 100 mg of leaf tissue were collected from the second youngest leaf of each genotype. The tissue was cut into 10 mm long, placed in 10 ml distilled deionized water, and incubated at room temperature for 2 h before autoclaving. The electrical conductance of the solution was measured before and after autoclaving for EC_initial_ and EC_final_ values, respectively. Since ion leakage could vary between genotypes, the index of salt injury was estimated with respect to the ion leakage of the corresponding genotype grown in control conditions, following the formula of Flint et al. (1967): Ion_leak = 100 (Rt-Ro)/(1-Ro); where Ion_leak is the index of injury by ion leakage; Ro = EC_initial_/EC_final_ of the control plant, and Rt = EC_initial_/EC_final_ of the stressed plant.

#### Chlorophyll concentration

Leaf yellowing was observed in plants 4 days after salinization. To compare the differences among genotypes, the relative chlorophyll concentration was measured nondestructively from the mid-part of the second youngest leaf in control and stressed rice genotypes using the SPAD 502 chlorophyll meter (Spectrum Technologies, Inc.) after 4 days. The relative percent reduction in chlorophyll concentration was computed by the formula: Chl_R = 100 (Chl_control_ − Chl_stress_/Chl_control_).

#### Growth parameters

Changes in shoot and root length in response to salinity stress were measured for each genotype 7 days post salinization (DPS). Shoot length was measured from the base of the plant to the tip of the longest leaf, while the root length was measured from the base of the plant to the tip of the root mass. To account for genotypic differences, all comparisons were done with respect to the control. Hence, the percent reduction in root and shoots were computed by a formula similar to the chlorophyll percent reduction.

#### Visual salt injury score (SIS)

Plant responses to salinity stress were evident 7 DPS. For visual scoring, the IRRI standard evaluation scoring was followed (Gregorio et al., 1997). The plant scored 3 if it showed little to no leaf damage, but was stunted compared to the corresponding genotype grown in the control solution. A score of 5 was given if the plant was stunted with green rolled leaves having a few whitish tips. A plant showing only green culm with dried leaves was scored 7, and a score of 9 was given if the plant was completely dead. All visual scoring was done when the sensitive check IR29 had a score of 7 or 9. The mean SIS score of each genotype was computed from 10 individual plants per experiment.

#### Na-K analysis

The concentration of sodium and potassium in the root and shoot were determined for each genotype grown in saline conditions after 7 days. Five plants per genotype were rinsed with distilled water and then dried for 2 days at 65°C. Each dried tissue was ground by mortar and pestle and 100 mg were digested with 5 ml of nitric acid and 3 ml hydrogen peroxide at 152–155°C for 3 h in a hood. The digested tissue was diluted to a final volume of 12.5 ml, and the concentration of sodium and potassium were quantified using a flame photometer (Jenway model PFP7). The estimated concentration was calculated from a standard curve. The absolute concentration was computed based on the dilution of the sample.

#### Statistical analyses

To evaluate the genotypic differences for each trait, ANOVA and comparison of means by Dunnett's test were conducted using the GLIMMIX procedure against IR29 or Pokkali. The genotype was entered as the fixed effect and the replication as a random effect. To improve the normality of the data for analysis of genotypic differences, values were anchored to 1, then log transformed prior to data analysis. Correlation among traits was computed using the CORR procedure of SAS Version 9.3 for Windows (SAS Institute Inc, 2011), based on the pooled least square (LS) mean of three replications per trait.

#### Clustering and discriminant analyses

To characterize the level of salinity tolerance of the 49 varieties, the LS mean values of genotypes for six traits (SIS, ion_leak, chlorophyll and shoot length reduction, shoot K concentration, and shoot Na^+^/K^+^ ratio) were used in multivariate cluster analysis of NTSYSpc 2.10 t (Rohlf, 2000). Because of different scaling and to give equal importance among the trait variables, the data were standardized to have a mean of 0 and a variance of 1. Euclidean distances between all pairs of genotypes were computed from standardized six seedling traits, and the phenogram of rice genotypes was constructed based on the UPGMA (Unweighted Pair Group Method with Arithmetic Mean). Based on the ranking of the group mean SIS, the clusters were classified as HT, tolerant (T), moderately tolerant (MT), sensitive (S), and highly sensitive (HS). To confirm the classification of genotypes, the same data for clustering were used in discriminant analyses with the group assignment for each genotype. The six seedling traits were considered as dependent variables, and the salinity clusters (HT, T, MT, S, and HS) were considered as independent variables. All genotypes were then given an equal prior probability to be grouped into the five levels of salinity tolerance. The PROC DISCRIM, PROC CANDISC, and the GLM procedures for MANOVA were run in SAS v9.3 (SAS Institute Inc, 2011) to determine the differences among the levels of salinity groupings.

### Genetic diversity analysis

Plants were grown in non-saline growth medium, and leaf tissues were harvested from a single plant of each genotype. The genomic DNA from each genotype was isolated following the CTAB method (Chen and Ronald, 1999). The DNA concentration was quantified by a spectrophotometer (NanoDrop ND-1000) and was adjusted to a final concentration of 25 ng/μl for PCR amplification.

One hundred forty-six SSR markers, evenly spaced across the 12 chromosomes of rice, were used in PCR amplification for genetic diversity (Supplementary Table S2). For each 25 μl reaction, the PCR mixture contained 12.8 μl water, 2.5 μl 10X PCR buffer, 2.5 μl 25 mM MgCl_2_, 2.5 μl 2 mM dNTPs, 1.25 μl 50 ng/μl reverse and forward primers, and 1U Taq polymerase (Promega Corporation, Madison, USA). The reactions were run for 35 cycles of 94°C for 45 s, 55°C for 45 s, and 72°C for 1 min with a final extension at 72°C for 5 min. The PCR products were analyzed by 4.5% SFR agarose gel electrophoresis. Four hundred twenty-seven alleles were then scored as 1 or 0 for the presence or absence of a PCR band. The pairwise distance matrix was computed among genotypes using the dice coefficient, and then used in tree construction by unweighted neighbor-joining as implemented in DARwin 6.0 (Perrier et al., 2003). AMOVA, genetic distance, and Mantel's test were performed using GenAlEx (Peakall and Smouse, 2012) to evaluate genetic diversity.

## Results

During the experiment, greenhouse temperature ranged between 24 and 29°C during the day. Plants assigned to control and the corresponding genotypes to salinity treatment grew uniformly after 2 weeks in non-saline hydroponic solution. Upon addition of sodium chloride at 12 dSm^−1^, most of the rice genotypes showed leaf rolling after 2 to 3 h. Growth of the plants stopped by the 2nd or 3rd day, followed by chlorosis and leaf bleaching from the tip of the leaf blade to the leaf base on the 4th or 5th day. By the 7th to 9th day post salinization, susceptible seedlings of IR29 were dead. Tolerant varieties also showed the same early response to salinity stress, but at 4th or 5th day, they showed some signs of recovery, such as leaf greening and growing of the youngest leaf.

Significant differences among genotypes were observed for some of the traits investigated (Table 1). However, the differences across genotypes were not significant in the root length reduction (RtL_R), root sodium concentration in (Rt_Na), root potassium concentration (Rt_K), root sodium: potassium ratio (Rt_Na/K), and shoot sodium concentration (Sh_Na) at *P* < 0.05 level of significance. For ion leakage, genotypic differences were highly significant (*P* < 0.0001). It ranged from 27 to 72%, indicating a wide variation in the membrane permeability across 49 genotypes under salt stress. The exotic donor cultivars from IRRI showed low ion leakage not greater than 42%, while IR29 had 52%. Among the USA varieties, salt tolerant lines were CL162, Cypress, and CL261, with ion leakage values of 33, 40, and 40%, respectively.

Percent reduction in chlorophyll concentration (Chl_R) among genotypes was highly significant (*P* < 0.0001). Pokkali had 35% reduction while IR29 had 52%. Among the donor genotypes from IRRI, CSR II had the lowest chlorophyll reduction of 4%. FL478, IR944-102-2-3-2, TCCP-266, and Geumgangbyeo had 18, 19, 24, and 26% chlorophyll reduction, respectively. Among the USA genotypes, Cheniere, R609, LAH10, Cypress, Neptune, Caffey, and Templeton showed less than 40% chlorophyll reduction.

At the 7th day post salinization, salt injury scores (SIS) were significantly different between genotypes (*P* < 0.0001). Pokkali had a mean SIS of 2.9 and IR29 had a score of 7.7. The donor genotypes showed varying levels of tolerance with SIS range between 2.9 and 6.1. The USA genotypes were sensitive, except for R609, LAH10, and Cypress with SIS of 4.4, 4.4, and 5.1, respectively. In addition, Cheniere, Roy J, Jupiter, Neptune, Caffey, Templeton, Taggert, and CL162 showed an intermediate response with SIS of 5.9–6.2. The rest of the USA genotypes were HS to salt stress with SIS more than 7.0.

Other morphological responses to salinity, such as root and shoot length, showed variation among genotypes. Root growth was inhibited in all genotypes, and the reduction was as high as 56%. However, analysis of variance for the percent root length reduction (RtL_R) did not show significant genotypic differences (*P* = 0.9927). In contrast, percent shoot length reduction (ShL_R) was highly significant (*P* < 0.0001) among genotypes. Pokkali and Hasawi had the lowest growth reduction (34%) while IR29 was reduced by 40%. All USA genotypes displayed shoot growth reduction that ranged from 44 to 58%, indicating the sensitivity of USA genotypes to salt stress.

The Na^+^ and K^+^ concentration were determined in roots and shoots of the 49 genotypes. All genotypes grown in salinized medium showed an increased Na^+^ concentration in roots and shoots, while the K^+^ concentration was reduced when compared to non-salinized condition (data not shown). Varying concentrations of Na^+^ were observed among the genotypes. In general, shoot Na^+^ concentration was about two times the concentration of Na^+^ in roots. Analysis of variance showed that neither root Na^+^ nor shoot Na^+^ concentration was significantly different among genotypes, despite the higher concentration of Na^+^ in susceptible IR29 than Pokkali. The genotypic differences in root K^+^ concentration were also not statistically significant (*P* = 0.3763) at 5% level of significance although the 49 genotypes showed differences in concentrations. In contrast, nearly non-significant genotypic differences for shoot K^+^ concentration was observed among genotypes (*P* = 0.0492). Donor genotypes from IRRI had shoot K^+^ concentrations that ranged from 900 to 1300 mmolkg^−1^. FL378 and Damodar had the highest shoot K^+^ concentration (1336 and 1333 mmolkg^−1^), while Pokkali and IR29 had shoot K^+^ concentrations of 995 and 821 mmolkg^−1^, respectively. On the other hand, all USA genotypes except Jazzman had low shoot K^+^ concentration ranging from 600 to 900 mmolkg^−1^. Examination of Na^+^/K^+^ ratio in root (Rt_Na/K) was not significant (*P* = 0.2619), but the shoot Na^+^/K^+^ ratio (Sh_Na/K) was significant (*P* = 0.0160) among genotypes. Donor cultivars and Geumgangbyeo had lower shoot Na^+^/K^+^ ratios compared to USA genotypes. IR29 had a Na^+^/K^+^ ratio of four while Pokkali had a ratio of 2.7. Interestingly, LAH10, which showed a SIS of 4.4, had a ratio of 2.9, while Cocodrie, CL162, Rex, Cheniere, LA0702085, and Jazzman-2 had shoot Na^+^/K^+^ ratios between 3.0 and 3.5.

### Correlation of traits related to salinity tolerance

To better understand the physiological traits that best describe salinity tolerance, relationships among all traits were analyzed (Table 2). Individual correlation of traits showed that SIS was positive and highly correlated to ion_leak, chlorophyll % reduction, shoot length % reduction, and shoot Na^+^/K^+^ ratio, but negatively correlated to shoot K^+^ concentration. The pattern of correlations was the same for shoot Na^+^/K^+^ ratio. Shoot Na^+^/K^+^ ratio was positive and highly correlated to ion_leak, chlorophyll % reduction, and shoot length % reduction; it was highly but negatively correlated to shoot K^+^ concentration. Shoot length reduction was also positive and highly correlated to ion_leak and chlorophyll reduction. Shoot K^+^ was negatively correlated to ion_leak and shoot length reduction, but significantly and positively correlated to shoot Na^+^ and root Na^+^. Root Na^+^/K^+^ ratio was positively correlated to root Na^+^ and negatively correlated to root K^+^. Taken together, ANOVA and correlation results indicated that SIS, ion leakage, chlorophyll reduction, shoot length reduction, shoot K^+^ concentration, and shoot Na^+^/K^+^ ratio are important parameters in defining the levels of salinity tolerance.

**Table 2:** **Pearson correlation matrix of seedling traits in response to salt stress at 12 dSm^−1^ in rice genotypes**.

	**SIS**	**RtL_R**	**Rt_Na**	**Rt_K**	**Rt_Na/K**	**Ion_leak**	**Chl_R**	**ShL_R**	**Sh_Na**	**Sh_K**	**Sh_Na/K**
SIS	1										
RtL_R	−0.006	1									
Rt_Na	0.0542	−0.136	1								
Rt_K	−0.123	−0.173	0.258	1							
Rt_Na/K	0.115	0.125	0.446^***^	−0.350^**^	1						
Ion_leak	0.474^***^	0.0689	−0.075	−0.184	0.105	1					
Chl_R	0.771^***^	0.0547	0.111	−0.128	0.208	0.289^*^	1				
ShL_R	0.538^***^	0.124	−0.233	−0.106	0.011	0.470^***^	0.442^***^	1			
Sh_Na	0.106	−0.338^*^	0.281	0.068	0.109	−0.138	0.257	−0.003	1		
Sh_K	−0.540^***^	−0.039	0.346^**^	0.222	0.083	−0.563^***^	−0.254	−0.435^***^	0.318^*^	1	
Sh_Na/K	0.644^***^	−0.208	−0.102	−0.265	0.038	0.473^***^	0.431^***^	0.373^**^	0.221	−0.746^***^	1

*SIS, salt injury score; Chl_R, % chlorophyll reduction; ShL_R, % shoot length reduction; RtL_R, % root length reduction; Ion_leak, index of injury by ion leakage; Rt_Na, root sodium concentration; Rt_K, root potassium concentration; Rt_Na/K, N/K ratio in root; Sh_Na, shoot sodium concentration; Sht_K, shoot potassium concentration; Sh_Na/K, Na/K ratio in shoot*.

* *Significant at the 0.05 probability level*.

** *Significant at the 0.01 probability level*.

*** *Significant at the 0.001 probability level*.

### Classification of 49 rice genotypes for salinity tolerance

**Figure 1:**
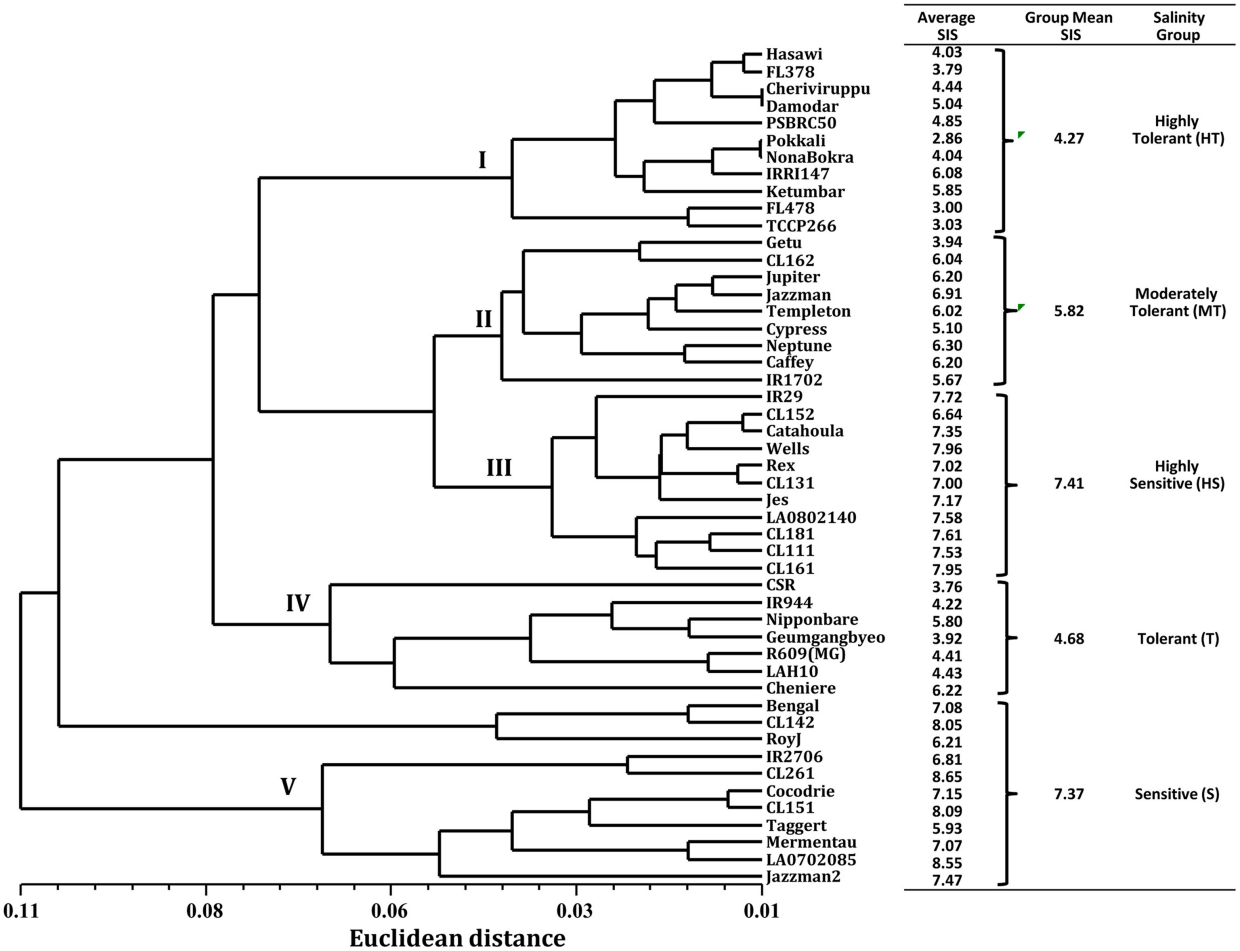
**Clustering of 49 genotypes by UPGMA based on Euclidean distance of six morphological and physiological trait responses to salinity stress**.

Because of the significant genotypic differences and high correlations in SIS, ion leakage, chlorophyll reduction, shoot length reduction, shoot K^+^ concentration, and shoot Na^+^/K^+^ ratio, we decided to use these parameters in the cluster analysis for the phenotypic classification of rice genotypes in response to salinity stress. The phenogram generated by UPGMA computed from the six traits (SIS, Ion_leak, Chl_R, ShL_R, Sh_K, and Sh_Na/K) produced five major clusters (Figure 1). From the ranking of their group SIS means, cluster I was assigned as HT, with the lowest group mean of 4.3. As expected, cluster I grouped the known HT genotypes such as Pokkali, Nona Bokra, FL478, TCCP266, FL378, Hasawi, and Cheriviruppu. Cluster II had a group SIS mean of 5.8 and was classified as moderately tolerant (MT). The USA genotypes such as CL162, Jupiter, Jazzman, Templeton, Cypress, Neptune, and Caffey grouped together in cluster II. The highest group SIS mean (7.4) was observed for cluster III and hence classified as HS. It included the sensitive check IR29 and 10 other USA genotypes. Cluster IV had a group SIS mean of 4.7 and was considered as tolerant (T) group, which contained CSRII, Nipponbare, Geumgangbyeo, R609, and LAH10. Cluster V was classified as sensitive (S) with a group SIS mean of 7.4, where popular genotypes such as Roy J, Cocodrie, Bengal, Mermentau, and Jazzman2 were placed.

The Fisher linear discriminant analysis (FLDA) is an approach similar to logistic regression, but the computation is more like the MANOVA or canonical correlation. The procedure initially computes the Mahalanobis distance of each genotype to a group and then uses it to classify the genotype into a group to which it has the smallest generalized squared distance (Truxillo, 2008). Results of FLDA indicated an error rate of 6.9%, owing to the three genotypes that were misclassified (Supplementary Table S3). IR1702, which was classified as moderately tolerant, should be placed in the tolerant group; Nipponbare should be classified as moderately tolerant instead of tolerant, and Jazzman2 should be grouped into the HS group instead of sensitive group. In FLDA, however, the test of homogeneity of covariance matrices was significant (*P* < 0.0001). Hence, we were prompted to use quadratic discriminant analysis (QDA) instead of FLDA. In QDA, the result indicated a 0% error rate, confirming that our genotype classification based on the clustering method was robust.

### Differentiation of salinity groups by canonical discriminant function and MANOVA

To further understand the grouping and to assess the extent of differences between salinity groups, canonical discriminant analysis was employed. Multivariate test statistics of nonlinear prediction of group membership based on the six physiological traits was highly significant in all statistics, thus confirming the likelihood of group membership prediction. Based on five groups and six trait variables, two canonical discriminant functions were high and significantly correlated for the prediction of genotype membership into salinity groupings. Canonical discriminant function 1 (Can1) and canonical discriminant function 2 (Can2) accounted for 81% and 12% of the variance in the traits, respectively (Supplementary Table S4). The loading of the variables to canonical discriminant functions showed that SIS, Chl_R, ShL_R, Ion_leak, and Sh_Na/K were positive and highly correlated to Can1, while Sh_K was negatively correlated (Supplementary Table S5). From the variance explained by Can1 and the loading of trait variables, it appeared that Can1 is a measure of the overall characteristics of salinity tolerance by the six parameters. In contrast, Can2 was positively correlated to Sh_K and Chl_R but negatively correlated to ShL_R and Ion_leak. Therefore, this result suggests that Can2 differentiates genotypes based on their K^+^ and chlorophyll concentrations. In Can1, the maximum separation of group means was observed between HT and S (−3.96 vs. 3.37) and mean separation between HS and T was 1.86 vs. −1.55. Examination of Can2 showed separation of HT from the T group (1.17 vs. −1.84) and separation of MT from the S group (−0.81 vs. 0.90). All groups with negative mean values to Can1 had some tolerance to salinity (HT, T, and MT). In contrast, HS and S groups had positive mean values to Can1.

**Figure 2:**
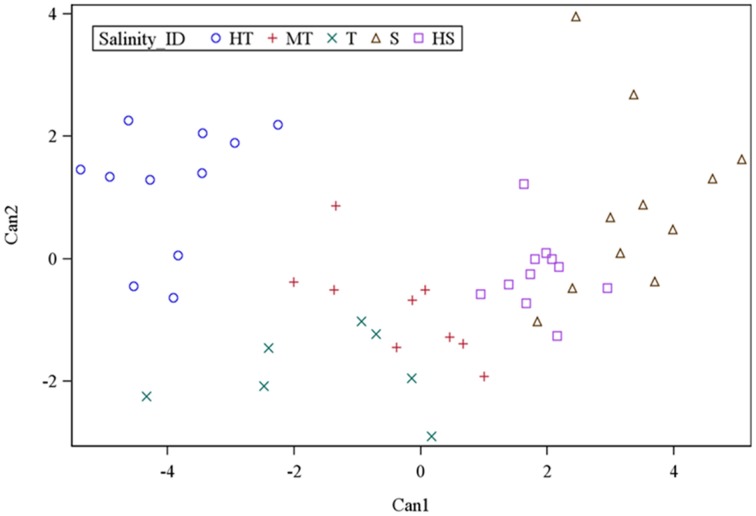
**Population structure of 49 rice genotypes by canonical discriminant analysis of morphological and physiological trait responses to salt stress**.

In the plot of salinity groups against Can1 and Can2, the MT group was placed in the center between the T and HS groups (Figure 2). The HT group had negative mean to Can1 (−3.96) and positive mean to Can2 (1.17), indicating that HT had low values in SIS, Ch_R, ShL_R, Ion_leak, and Sh_Na/K but with positive high K^+^ concentration. The T group had both negative mean values to Can1 (−1.55) and Can2 (−1.84), indicating that the T group is like the HT group, but it has lower K^+^ concentration as compared to HT group. Between T and MT, the T has higher negative mean values in both Can1 and Can2. The Sensitive (S) group had positive mean values to Can1 (3.37) and Can2 (0.90), indicating higher mean values in all traits and low K^+^ concentration. The HS group was the total opposite of HT group, with positive and negative mean values in Can1 (1.86) and Can2 (−0.23), respectively.

Further analysis by multivariate analysis of variance (MANOVA) for 6 variable traits across 5 groups indicated that the groups are significantly different. Moreover, LS means comparison for each trait between groups showed significant differences of HT from S and HS groups in all traits (Table 3, Supplementary Table S6). Conversely, the T group was significantly different from the HT group in ShL_R, ion leakage, and Sh_K, while a significant difference was observed only in Chl_R between T and MT. On the other hand, the S group was significantly different to MT in SIS, Chl_R, and ion leakage; and significantly different to HS in Chl_R alone. Nonetheless, overall pairwise contrasts between groups were highly significant in all comparisons, indicating the complete separation between groups based on the six quantitative traits.

**Table 3:** **Least square (LS) means of salinity groups in six parameters**.

**Group**	**SIS**	**Chl_R**	**ShL_R**	**Ion_leak**	**Sh_K**	**Sh_Na/K**
HT	4.27	32.84	39.58	32.98	1111.67	2.57
T	4.68	24.59	46.27	53.89	838.97	3.20
MT	5.82	42.18	48.93	44.57	878.65	3.60
S	7.37	68.63	47.59	55.68	797.46	3.92
HS	7.41	54.52	49.04	49.39	785.04	3.83

*SIS, salt injury score; Chl_R, % reduction in chlorophyll; ShL_R, shoot length % reduction; Ion_leak, index of injury by ion leakage; Sht_K, shoot potassium concentration; Sh_Na/K, Na/K ratio in shoot; HT, highly tolerant; T, tolerant; MT, moderately tolerant; S, sensitive; HS, highly sensitive*.

### Genetic diversity of 49 rice genotypes

**Figure 3:**
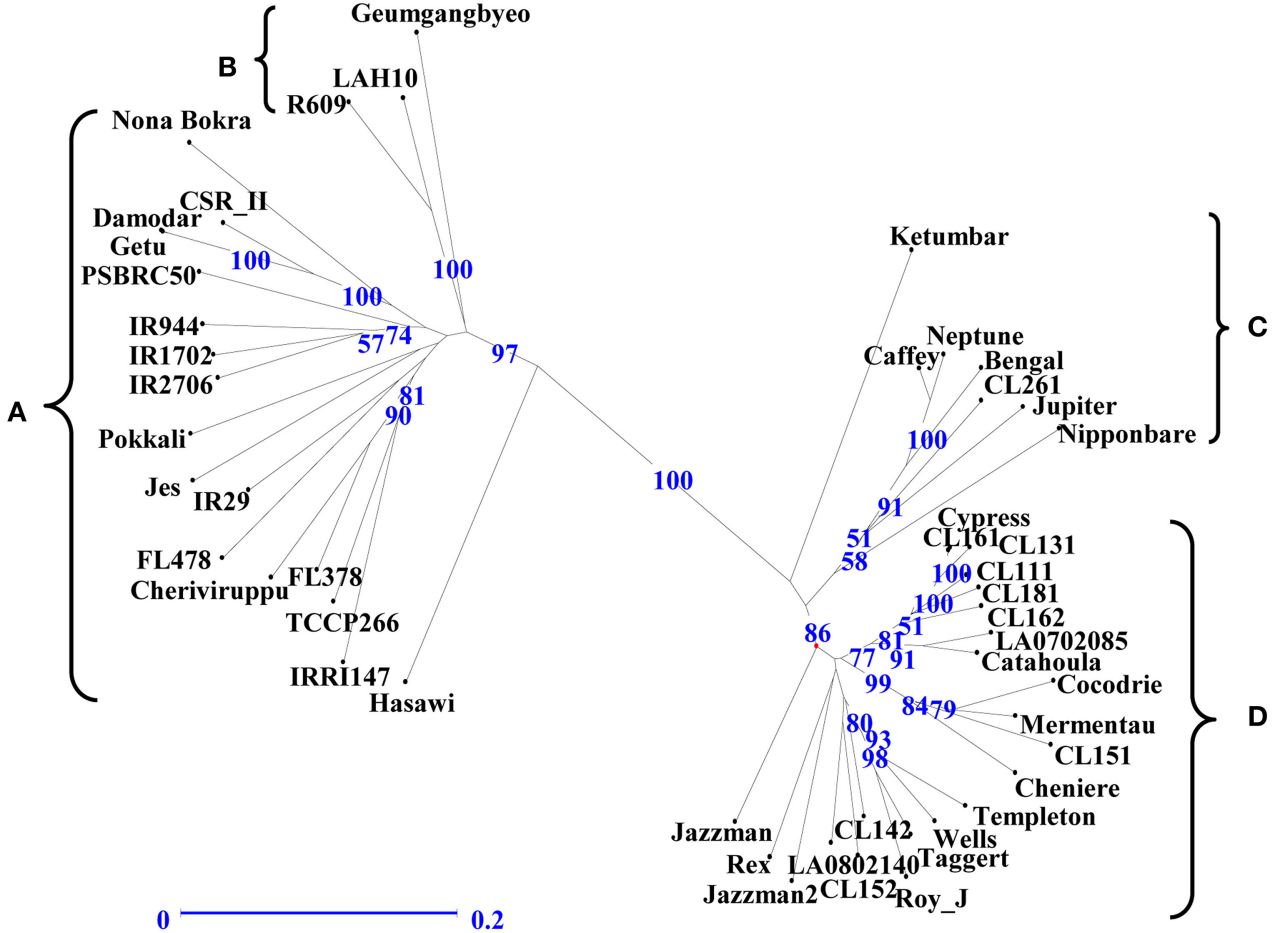
**Genotypic clustering by unweighted neighbor-joining tree showing the genetic relationship among the 49 rice genotypes based on 146 SSR markers**. Horizontal bar indicates distance by dice coefficient. Numbers on nodes are bootstrap values based on 100 iterations.

The genetic relationship among the genotypes was assessed to identify parental genotypes for the breeding program and to determine if the observed clustering of 49 genotypes based on salinity stress responses can be explained by their DNA profile. An unweighted neighbor-joining tree of 49 genotypes, based on 427 alleles using 146 SSR markers, separated the genotypes into two major groups of *indica* (clusters A, B) and *japonica* (clusters C, D) subspecies with two sub-clusters within a group (Figure 3). Analysis of molecular variance showed significant genetic differences among the four populations [PhiPT = 0.505 at P (rand perm. 999) =0.001] with 49% and 51% variance within and among populations, respectively (Table 4). Differentiation of the clusters showed that USA varieties had fewer numbers of alleles, lower percentages of polymorphic loci and very few unique alleles compared to *indica* genotypes. Based on Shannon's information index, the donor genotypes (*indica* group) showed higher genetic diversity than the USA genotypes even with fewer sample sizes (Table 5). Similarly, Nei's genetic distance between the C and D clusters is only 0.093, indicating a narrow genetic diversity among the USA genotypes. The relationship between the subgroups among the USA varieties is the obvious separation of the medium grain (C) from the long grain varieties (D). Further examination of *indica* varieties showed that cluster A is a mixture of traditional and Pokkali-derived lines of medium and long grain cultivars. As expected, the aromatic rice variety ‘Jes’ (Anonymous, 2009), a long grain mutant of Khao Dawk Mali developed for temperate rice growing areas in the US, was grouped to cluster A. In contrast, Ketumbar, a short grain *indica* genotype from Indonesia (Negrão et al., 2011), was grouped into cluster C of medium grain *japonica* varieties. However, the grouping of tolerant Pokkali and susceptible IR29 in cluster A indicated that genetic profiling based on the SSR markers spanning the 12 chromosomes of rice cannot explain the varietal grouping based on salinity responses. Furthermore, the Mantel test of correlation between phenotypic and genetic distance matrices was low (*r* = 0.206) although significant at 999 permutation test. Therefore, the clustering suggests genetic similarity of genotypes based on subspecies and grain morphology.

**Table 4:** **Summary of analysis of molecular variance (AMOVA)**.

**Source of variation**		**df**	**SS**	**MS**	**Est. Variance**	**% Variance**
Among populations		3	1630.032	543.344	46.600	51%
Within populations		45	2054.132	45.647	45.647	49%
Total		48	3684.163		92.247	100%
PhiPT:	0.505					
P(rand perm. 999)	0.001					

*Populations refer to the rice clusters (A, B, C, and D) in Figure 3. Df, degree of freedom; SS, sum of squares; MS, mean square; Est. Variance, estimated variance; % Variance, percent variance; PhiPT, estimate of genetic distance among populations; P (rand perm.999), significance of genetic distance at 999 random permutations*.

**Table 5:** **Genetic differentiation between population clusters of rice genotypes by 146 SSR markers**.

**Population cluster**	**A**	**B**	**C**	**D**
Sample size	17	3	7	22
Mean No. of different alleles	1.618	0.883	1.199	1.164
Mean No. of effective alleles = 1/(p^∧^2 + q^∧^2)	1.378	1.237	1.291	1.264
Mean expected heterozygosity = 2 ^*^ p ^*^ q	0.227	0.133	0.173	0.157
Shannon's Information Index = −1^*^ (p ^*^ Ln (p) + q ^*^ Ln(q))	0.35	0.194	0.263	0.239
No. of different bands	358	237	284	277
No. of bands unique to a single population	40	2	6	5
Percentage of polymorphic loci	78%	33%	53%	52%

*Population clusters (A, B, C, and D) are from the Figure 3*.

## Discussion

Crop breeding programs aim to make new varieties that will better cope with abiotic and biotic stresses. In developing salt tolerant cultivars, rice breeding programs are making efforts to evaluate diverse germplasm to enhance their utility (Ismail et al., 2007). Overall, the *indica* cultivars are more tolerant to salinity than japonica cultivars because of their superior ability in excluding Na^+^, absorbing K^+^, and maintaining a low Na^+^/K^+^ ratio in shoots (Gregorio and Senadhira, 1993; Lee et al., 2003). Many traditional landraces that can withstand high levels of salinity are good candidates for breeding salt-tolerant cultivars. However, due to their undesirable agronomic traits, they are not used (Gregorio et al., 2002). In the USA, rice breeding programs in the Southeastern region have been successful in breeding high yielding varieties. However, none of these varieties have been evaluated for the level of tolerance to salinity stress. Here, we evaluated the genetic diversity, as well as the morphological and physiological responses, of 49 diverse rice genotypes that included rice cultivars of the Southeastern USA and several exotic donors and breeding lines with varying levels of tolerance to salinity stress. The six quantitative traits were used for objective varietal classification and delineation of the levels of salinity tolerance. The use of cluster analysis and validation by discriminant analysis was implemented for accurate classification for salinity tolerance.

Among the traits evaluated for salt stress response, genotypes varied significantly for shoot parameters, but not for root traits (Table 1), suggesting that salinity tolerance is more likely controlled in the shoot. This possibly explained the higher occurrence of induced DNA methylation in shoots as compared to roots in some rice varieties tested for salinity response (Karan et al., 2012). Different trait parameters showed different ranking of genotypes in response to salinity stress, indicating wide natural phenotypic variation among the 49 rice genotypes. The correlation of all traits allowed us to identify relationships among traits that described salinity tolerance. Instead of considering only visual SISs, other parameters, such as ion leakage, chlorophyll concentration, shoot length, shoot K^+^ concentration, and shoot Na^+^/K^+^ ratio could be unbiased parameters for assessing salinity tolerance.

Previous studies suggested that the toxicity of salt stress could be due to Na^+^ accumulation in the shoot (Lin et al., 2004). Our results, however, did not show that sodium accumulation was more in salt-sensitive varieties, which could lead to increased ion leakage due to injured plasma membranes (Lv et al., 2012). Instead, our results are similar to the findings of Yeo et al. (1990), in which there was no significant variation among rice genotypes in the shoot uptake of sodium. Likewise, we did not find a significant correlation of visual SIS and shoot sodium concentration (Table 2). These results suggested that salinity tolerance among the tolerant varieties is not a function of restricting sodium uptake, but more likely in the compartmentalization of sodium to alleviate its toxic effect (Blumwald, 2000). This finding is consistent with prior reports in rice cv. Pokkali (Kader and Linberg, 2005), *Salicornia europaea* (Lv et al., 2012), *Arabidopsis thaliana* (Apse et al., 1999), and *Saccharomyces cerevisiae* (Nass and Rao, 1988). Other donors for a high degree of salt tolerance as Pokkali were FL478, FL378, TCCP266, Nona Bokra, Hasawi, Damodar, and Cheriviruppu (Group I, Figure 1). The high positive correlation of shoot length reduction and % chlorophyll reduction to SIS indicated that the photosynthetic capacity of salt-sensitive plants became limited, leading to chlorosis and shoot growth reduction under salt stress (Apse et al., 1999; Lin et al., 2004; Munns and Tester, 2008). Among the donor cultivars, Hasawi and Pokkali had the least growth reduction and relatively low chlorophyll reduction. In addition, CSR II, FL478, TCCP 266, IR944, and Geumgangbyeo showed low chlorophyll reduction despite high shoot growth reduction.

Another obvious trait for the mechanism of tolerance among the donor cultivars is the high potassium uptake resulting in lower Na^+^/K^+^ ratio (Gregorio and Senadhira, 1993; Koyama et al., 2001; Bonilla et al., 2002; Ren et al., 2005; Pushparajan et al., 2011; Wang et al., 2012). In contrast, the USA varieties, with the exception of Jupiter and Jazzman, had shoot K^+^ concentrations less than 900 mmolkg^−1^. Previous studies by Ren et al. (2005) indicated that the *SKC1* gene from Nona Bokra maintains high shoot K^+^ concentration, thereby regulating the Na^+^/K^+^ homeostasis under salt stress. Our results showed that, aside from Nona Bokra, other donor cultivars that can be used for improvement of salinity tolerance through high shoot K^+^ concentration and low Na^+^/K^+^ ratio are FL378, Damodar, Hasawi, Ketumbar, PSBRC50, Cheriviruppu, and IR2706-11-2.

Previous attempts to characterize salt-tolerant rice varieties were done using agro-morphological traits (Caldo et al., 1996; Zeng et al., 2003; Sanni et al., 2012). In most breeding strategies, the simple visual salt injury scoring (Gregorio et al., 1997) is widely used for characterization because it reflects the overall plant's response to salt stress. However, the inherent subjectivity and the quantitative nature of salinity tolerance complicate the evaluation for salinity tolerance. Thus, other studies suggest the use of Na-Ca selectivity (Zeng et al., 2003), tiller number and Na-K selectivity (Zeng, 2005), and proline concentration (Kanawapee et al., 2012) as criteria for classification of rice varieties for salt tolerance. However, varietal differences showed that it is natural for varieties to be superior in one trait and inferior in others (Yeo et al., 1990). Instead of characterizing rice genotypes for traits one by one, we employed the multivariate cluster analysis using the six quantitative traits across the 49 genotypes. The five traits (ShL_R, Chl_R, ion leakage, Sh_K, and Sh_Na/K) showed significant and high correlation to SIS. Thus, they are unbiased estimate of a variety's performance in response to salinity stress. Our results demonstrated that the groupings were robust, and varietal assignment to the level of salinity tolerance was confirmed by discriminant analysis. As indicated by MANOVA and discriminant functions, the levels of salinity tolerance were significantly distinct against each other. The morphological responses of the HT group were least affected by salt stress due to high K^+^ uptake, resulting in low Na^+^/K^+^ ratios and possibly by effective compartmentalization of Na^+^ in shoot. In contrast, higher shoot length reduction, higher ion leakage, and lower shoot K^+^ concentration separated the T group from HT varieties. The T and MT groups had the same salt responses, but the ability to maintain lower chlorophyll reduction made T superior to MT. The HT group was significantly superior to the S and HS groups in all traits, while the T and MT groups were statistically superior to S and HS only in the overall visual score and chlorophyll reduction. Therefore, the genotypes in T and MT groups offered a novel source of tolerance and an apparent mechanism distinct from those found in the HT group. Between S and HS, trait responses were not significantly different, except in chlorophyll reduction. The S group had even higher chlorophyll reductions than HS group, suggesting that S and HS should be treated as one group (Table 3 and Supplementary Table S6). While SIS offers a simple screening method and accounted for the overall performance of rice varieties under salt stress, our results emphasized the importance of five other traits (ShL_R, Chl_R, ion leakage, Sh_K, and Sh_Na/K) in objective varietal classification for salinity tolerance. Furthermore, our results demonstrated the power of multivariate analyses (clustering, MANOVA, and canonical and linear discriminant analyses) in confirmation and demarcation of levels of tolerance. Overall, the phenotypic clustering indicated the absence of HT USA varieties. However, LAH10, R609, and Cheniere exhibited some level of tolerance. LAH10 is a rice hybrid developed from R609. Thus, it is likely that the tolerance of LAH10 is inherited from R609.

Another important finding in this study is the information on genetic diversity. Numerous studies have classified rice varieties using DNA based markers such as RFLP (Zhang et al., 1992), AFLP (Subudhi et al., 1998), SSR (Ni et al., 2002), and SNP markers (McNally et al., 2009). Similar to previous differentiation studies using DNA markers (Zhang et al., 1992; Ni et al., 2002; Thomson et al., 2007), the genotypic grouping mainly separated the genotypes into japonica or indica subspecies (Figure 3). Among the USA genotypes, LAH10 and R609 clustered into the indica group, thus confirming the absence of high tolerance among the USA japonica varieties. Overall, despite the use of 146 markers resulting to 427 scored alleles, genotypic clustering was independent of phenotypic clustering in response to salt stress. Our results were consistent with the findings of Zeng et al. (2004), who used only 25 SSR markers to evaluate genetic diversity among rice genotypes with different adaptations to saline soils. The genotypic clustering separated the *indica* from the *japonica* clades, but not on the basis of salinity response. Interestingly, the 49 genotypes were subdivided into either long grain or short grain. Therefore, our results suggested a limitation of whole genome scanning using SSR markers in differentiating the polymorphism between salt tolerant and sensitive lines. Since salinity tolerance is polygenic in nature, it is likely that the markers we used have little or no association at all to the genes controlling salt tolerance. As genotyping by sequencing is becoming more accessible, it is likely the best way to increase the resolution of genetic differentiation that eventually can aid in genomic selection or development of markers linked to the physiological traits for salinity tolerance. Those markers will be useful in the marker-assisted breeding for pyramiding of physiological traits contributing to high tolerance (Yeo and Flowers, 1986). Nonetheless, the result of our DNA profiling indicated a narrow genetic diversity among USA varieties and therefore emphasized the need to expand the gene pool of USA rice germplasm, particularly for abiotic stress tolerance through the use of *indica* germplasm. Our results confirmed that exotic germplasm such as Nona Bokra, Hasawi, Cheriviruppu, Damodar, Ketumbar, Pokkali, TCCP266, FL378, and FL478 (Cluster I) possess high salinity tolerance during the seedling stage. However, many of these genotypes are photosensitive. Our initial salinity screening during the reproductive stage (data not shown) showed high grain sterility among the non-photosensitive donor cultivars except the TCCP266 genotype. TCCP266 is a somaclonal variant of Pokkali with better agronomic traits and with white pericarp (Gregorio et al., 2002). In contrast, Geumgangbyeo, LAH10, and R609 (Cluster IV-tolerant group) showed less sterility and less grain weight reduction during reproductive stage screening. While access to genetic diversity is an important component to a successful breeding strategy (Negrão et al., 2011), our results showed that the USA varieties were genetically more distant to cluster B (Figure 3). Therefore, Geumgangbyeo, R609, and LAH10 can be used as novel sources of seedling and reproductive salinity tolerance. Geumgangbyeo is a semi-dwarf rice variety from South Korea, and it is listed as a salt tolerant cultivar during the seedling stage in the GRIN database (http://www.ars-grin.gov/cgi-bin/npgs/html/ob2_acc.pl?75019+5.04+5.6, accessed 2 October 2014). Our results showed that it has a SIS of 3.92, lower root length reduction, higher chlorophyll content, lower shoot Na^+^ concentration, and lower Na/K ratio relative to Pokkali. LAH10 is a medium grain hybrid rice developed from R609 that is a restorer line used in hybrid rice breeding. Therefore, the use of R609 or LAH10 will enhance the prospect of developing salt tolerant hybrid rice.

Overall, our study demonstrated the use of several multivariate analyses in the classification and validation of the differences among rice genotypes for salinity tolerance. Effective identification and selection for high tolerance can be achieved by the accumulation of multiple favorable traits under salt stress. Thus, we propose the use of a linear combination of multiple traits as a predictor of tolerance for unbiased classification. Finally, the rice genotypes identified here will provide novel sources of salinity tolerance at the seedling stage.

### Conflict of interest statement

The authors declare that the research was conducted in the absence of any commercial or financial relationships that could be construed as a potential conflict of interest.

## Abbreviations

DPS: Days post salinization
FLDA: Fisher linear discriminant analysis
GRIN: Germplasm Resources Information Network
HS: Highly susceptible
HT: Highly tolerant
MANOVA: Multivariate analysis of variance
MT: Moderately tolerant
SIS: Salt injury score
SSR: simple sequence repeat
S: Susceptible
T: Tolerant
UPGMA: Unweighted pair group method with arithmetic mean
QDA: Quadratic discriminant analysis.
