# Supplementary material for: Genetic variation in Southern USA rice genotypes for seedling salinity tolerance
Source: Front Plant Sci. 2015 May 27;6:374. doi: 10.3389/fpls.2015.00374 (PMC4444739; doi:10.3389/fpls.2015.00374)
Supplement: Supplementary file 1 [file Table1.DOCX]

**Supplementary Tables**

Suppl. Table S1 List of genotypes used in the experiment, their source, and some key agronomic attributes.

| Genotype | Source^#^ | Subspecies | Photosensitivity | Presence of awn | Grain type | Pericarp color |
| --- | --- | --- | --- | --- | --- | --- |
| Hasawi (IRGC 16817) | IRRI Genebank | Indica | no | awned | medium grain | red |
| Cheriviruppu | IRRI Genebank | Indica | yes | awned | medium grain | red |
| Pokkali (IRGC 108921) | IRRI Genebank | Indica | yes | awned | medium grain | red |
| Nona Bokra (IRGC 01231) | IRRI Genebank | Indica | yes | no | medium grain | red |
| FL478 | IRRI Genebank | Indica | no | awned | long grain | red |
| FL378 | IRRI Genebank | Indica | yes | awned | long grain | red |
| TCCP-266-1-38-13-1-3 | IRRI Genebank | Indica | no | awned | long grain | white |
| IRRI 147 | IRRI Genebank | Indica | no | no | medium grain | white |
| Ketumbar (IRGC 13516) | IRRI Genebank | Indica | yes | no | short grain | white |
| Damodar (IRGC 17038) | IRRI Genebank | Indica | yes | no | medium grain | white |
| Getu (IRGC 17041) | IRRI Genebank | Indica | yes | no | medium grain | white |
| CSR II (IRGC 83240) | IRRI Genebank | Indica | no | no | medium grain | white |
| PSBRC50 (IRGC 99706) | IRRI Genebank | Indica | no | no | long grain | white |
| IR 1702-74-3-2 (PI 399813) | GRIN | Indica | yes | awned | long grain | white |
| IR 944-102-2-3-2 (PI 408628) | GRIN | Indica | no | awned | long grain | white |
| IR 2706-11-2 (PI 408508) | GRIN | Indica | no | no | long grain | white |
| Nipponbare (GSOR# 70) | USDA (Arkansas) | Japonica | no | no | medium grain | white |
| Geumgangbyeo | GRIN | Indica | no | no | medium grain | white |
| IR29 (IRGC 30412) | IRRI Genebank | Indica | no | no | long grain | white |
| Cocodrie | LRRS | Japonica | no | no | long grain | white |
| R609 (MG) | LRRS | Indica | no | no | medium grain | white |
| LAH 10 | LRRS | Indica | no | no | long grain | white |
| LA 0802140 | LRRS | Japonica | no | no | long grain | white |
| Cheniere | LRRS | Japonica | no | no | long grain | white |
| Bengal | LRRS | Japonica | no | no | medium grain | white |
| CL 152 | LRRS | Japonica | no | no | long grain | white |
| Roy J | LRRS | Japonica | no | awned^$^ | long grain | white |
| Rex | LRRS | Japonica | no | awned^$^ | long grain | white |
| CL142 | LRRS | Japonica | no | awned^$^ | long grain | white |
| Mermentau | LRRS | Japonica | no | no | long grain | white |
| Jupiter | LRRS | Japonica | no | no | medium grain | white |
| Wells | LRRS | Japonica | no | awned^$^ | long grain | white |
| Catahoula | LRRS | Japonica | no | no | long grain | white |
| CL151 | LRRS | Japonica | no | no | long grain | white |
| Jazzman | LRRS | Japonica | no | awned^$^ | long grain | white |
| Neptune | LRRS | Japonica | no | no | medium grain | white |
| Caffey | LRRS | Japonica | no | no | medium grain | white |
| Templeton | LRRS | Japonica | no | awned^$^ | long grain | white |
| Taggert | LRRS | Japonica | no | awned^$^ | long grain | white |
| Jazzman-2 | LRRS | Japonica | no | awned^$^ | long grain | white |
| Jes | LRRS | Indica | no | awned^$^ | long grain | white |
| CL162 | LRRS | Japonica | no | awned^$^ | long grain | white |
| CL181 | LRRS | Japonica | no | no | long grain | white |
| CL111 | LRRS | Japonica | no | no | long grain | white |
| CL131 | LRRS | Japonica | no | no | long grain | white |
| Cypress | LRRS | Japonica | no | no | long grain | white |
| CL161 | LRRS | Japonica | no | no | long grain | white |
| LA 0702085 | LRRS | Japonica | no | no | long grain | white |
| CL261 | LRRS | Japonica | no | no | medium grain | white |

^#^ GRIN, Germplasm Resources Information Network; LRRS, LSU Agricultural Center Rice Research Station

^$^ These cultivars developed short awns in greenhouse conditions in this study.
