# Supplementary material for: Genetic variation in Southern USA rice genotypes for seedling salinity tolerance
Source: Front Plant Sci. 2015 May 27;6:374. doi: 10.3389/fpls.2015.00374 (PMC4444739; doi:10.3389/fpls.2015.00374)
Supplement: Supplementary file 2 [file Table2.DOCX]

Suppl. Table S2 List of SSR markers and allele variations across 49 rice genotypes.

| SSR marker | Chromosome | Map Position (Mb)^1^ | No. of Allele | PIC value |
| --- | --- | --- | --- | --- |
| RM220 | 1 | 4.4 | 2 | 0.498 |
| RM283 | 1 | 4.8 | 2 | 0.245 |
| RM6277 | 1 | 5.7 | 2 | 0.209 |
| RM10483 | 1 | 7.7 | 2 | 0.495 |
| RM1287 | 1 | 10.8 | 4 | 0.653 |
| RM8094 | 1 | 11.2 | 5 | 0.609 |
| RM3412 | 1 | 11.5 | 5 | 0.399 |
| RM10748 | 1 | 11.7 | 5 | 0.656 |
| RM140 | 1 | 12.3 | 2 | 0.040 |
| RM10793 | 1 | 12.5 | 4 | 0.519 |
| RM493 | 1 | 12.8 | 4 | 0.603 |
| RM10825 | 1 | 13.3 | 2 | 0.390 |
| RM10852 | 1 | 13.9 | 2 | 0.040 |
| RM10864 | 1 | 14.2 | 3 | 0.566 |
| RM562 | 1 | 14.6 | 5 | 0.429 |
| RM10890 | 1 | 14.7 | 2 | 0.266 |
| RM7075 | 1 | 15.1 | 5 | 0.604 |
| RM6711 | 1 | 16.1 | 3 | -0.090 |
| RM466 | 1 | 17.2 | 3 | -0.027 |
| RM9 | 1 | 23.3 | 3 | 0.521 |
| RM5 | 1 | 23.9 | 3 | 0.452 |
| RM2318 | 1 | 24.1 | 3 | 0.919 |
| RM8129 | 1 | 25.0 | 3 | 0.612 |
| RM3143 | 1 | 26.8 | 3 | -0.517 |
| RM1297 | 1 | 28.6 | 2 | 0.475 |
| RM5389 | 1 | 35.7 | 3 | 0.561 |
| RM5781 | 1 | 35.7 | 2 | 0.300 |
| RM8278 | 1 | 36.6 | 4 | 0.686 |
| RM315 | 1 | 36.7 | 2 | 0.425 |
| RM5362 | 1 | 41.0 | 2 | 0.039 |
| RM3362 | 1 | 43.0 | 2 | 0.490 |
| RM84 | 1 | na | 2 | 0.307 |
| RM23 | 1 | na | 4 | -0.392 |
| RM154 | 2 | 1.1 | 8 | -1.434 |
| RM262 | 2 | 20.7 | 3 | 0.599 |
| RM263 | 2 | 25.8 | 3 | 0.466 |
| RM221 | 2 | 27.6 | 2 | 0.498 |
| RM221 | 2 | 27.6 | 4 | 0.563 |
| RM250 | 2 | 32.7 | 3 | 0.533 |
| RM29 | 2 | na | 2 | 0.479 |
| RM266 | 2 | na | 2 | 0.483 |
| RM3203 | 3 | 0.8 | 3 | 0.556 |
| RM5474 | 3 | 3.8 | 2 | 0.483 |
| RM5819 | 3 | 4.2 | 3 | 0.566 |
| RM5480 | 3 | 5.3 | 2 | 0.475 |
| RM5513 | 3 | 6.2 | 2 | 0.348 |
| RM282 | 3 | 12.4 | 2 | 0.398 |
| RM6080 | 3 | 13.9 | 2 | 0.483 |
| RM8208 | 3 | 22.4 | 5 | 0.556 |
| RM3525 | 3 | 30.4 | 4 | 0.670 |
| RM3564 | 3 | 33.4 | 2 | 0.459 |
| RM6084 | 3 | 33.5 | 3 | 0.263 |
| RM7389 | 3 | 36.1 | 3 | 0.338 |
| RM7 | 3 | na | 3 | 0.716 |
| RM571 | 3 | na | 3 | 0.591 |
| RM5633 | 4 | 13.1 | 3 | 0.598 |
| RM3742 | 4 | 19.7 | 2 | 0.425 |
| RM5979 | 4 | 20.8 | 2 | 0.300 |
| RM3866 | 4 | 23.2 | 3 | 0.522 |
| RM3839 | 4 | 23.9 | 3 | 0.348 |
| RM1388 | 4 | 25.0 | 4 | 0.633 |
| RM3288 | 4 | 27.4 | 3 | 0.566 |
| RM317 | 4 | 29.0 | 2 | 0.498 |
| RM5503 | 4 | 30.1 | 2 | 0.408 |
| RM3836 | 4 | 31.6 | 3 | 0.979 |
| RM348 | 4 | 32.6 | 2 | 0.479 |
| RM5506 | 4 | 33.3 | 3 | 0.533 |
| RM127 | 4 | 34.5 | 4 | 0.386 |
| RM5579 | 5 | 0.5 | 3 | 0.608 |
| RM5361 | 5 | 0.5 | 2 | 0.384 |
| RM159 | 5 | 0.8 | 6 | -0.143 |
| RM1366 | 5 | 2.9 | 4 | 0.336 |
| RM3419 | 5 | 5.3 | 3 | 0.892 |
| RM289 | 5 | 7.8 | 3 | 0.426 |
| RM6645 | 5 | 15.0 | 2 | 0.179 |
| RM5454 | 5 | 17.8 | 3 | 0.541 |
| RM146 | 5 | 18.0 | 4 | 0.639 |
| RM3663 | 5 | 21.3 | 2 | 0.150 |
| RM3616 | 5 | 26.8 | 2 | 0.440 |
| RM161 | 5 | na | 2 | 0.475 |
| RM469 | 6 | 0.6 | 2 | 0.476 |
| RM190 | 6 | 1.7 | 2 | 0.490 |
| RM225 | 6 | 3.4 | 3 | 0.679 |
| RM276 | 6 | 6.2 | 3 | 0.616 |
| RM4924 | 6 | 18.5 | 4 | 0.449 |
| RM7193 | 6 | 20.2 | 3 | 0.489 |
| RM6298 | 6 | 23.7 | 2 | 0.396 |
| RM5371 | 6 | 25.8 | 2 | 0.459 |
| RM6782 | 6 | 26.0 | 2 | 0.372 |
| RM295 | 7 | 0.4 | 5 | 0.287 |
| RM6663 | 7 | 2.1 | 2 | 0.319 |
| RM6574 | 7 | 4.6 | 2 | 0.449 |
| RM11 | 7 | 19.2 | 2 | 0.313 |
| RM11 | 7 | 19.2 | 4 | 0.601 |
| RM5508 | 7 | 23.5 | 3 | 0.379 |
| RM351 | 7 | 23.9 | 2 | 0.466 |
| RM134 | 7 | 26.6 | 4 | 0.671 |
| RM3555 | 7 | 27.9 | 3 | 0.603 |
| RM248 | 7 | 29.3 | 4 | 0.697 |
| RM10 | 7 | na | 2 | 0.439 |
| RM346 | 7 | na | 3 | 0.561 |
| RM6810 | 7 | na | 4 | 0.644 |
| RM408 | 8 | 0.1 | 4 | 0.634 |
| RM6863 | 8 | 2.0 | 2 | 0.506 |
| RM1376 | 8 | 3.2 | 3 | 0.573 |
| RM515 | 8 | 20.3 | 4 | 0.514 |
| RM195 | 8 | 21.4 | 3 | 0.433 |
| RM150 | 8 | 25.2 | 2 | 0.500 |
| RM3496 | 8 | 27.8 | 3 | 0.525 |
| RM8219 | 9 | 1.5 | 1 | 0.673 |
| RM566 | 9 | 14.7 | 2 | 0.594 |
| RM3700 | 9 | 15.4 | 3 | 0.628 |
| RM257 | 9 | 17.7 | 3 | 0.399 |
| RM160 | 9 | 19.8 | 2 | 0.475 |
| RM107 | 9 | 20.0 | 2 | 0.475 |
| RM6707 | 9 | 22.3 | 3 | -0.517 |
| RM205 | 9 | 22.7 | 1 | 0.833 |
| RM285 | 9 | na | 2 | 0.425 |
| RM219 | 9 | na | 3 | 0.646 |
| RM216 | 10 | 5.0 | 3 | 0.619 |
| RM8201 | 10 | 13.7 | 2 | 0.466 |
| RM258 | 10 | 18.0 | 2 | 0.480 |
| RM3451 | 10 | 21.5 | 2 | 0.499 |
| RM228 | 10 | 22.2 | 3 | 0.622 |
| RM333 | 10 | 22.3 | 3 | 0.766 |
| RM244 | 10 | na | 1 | 0.880 |
| RM269 | 10 | na | 3 | 0.106 |
| RM167 | 11 | 4.1 | 2 | 0.307 |
| RM202 | 11 | 9.0 | 3 | 0.553 |
| RM229 | 11 | 18.4 | 3 | 0.680 |
| RM1341 | 11 | 19.7 | 4 | -0.098 |
| RM206 | 11 | 22.0 | 4 | 0.681 |
| RM224 | 11 | 26.8 | 4 | 0.698 |
| RM21 | 11 | na | 2 | 0.408 |
| RM254 | 11 | na | 3 | 0.491 |
| RM3483 | 12 | 1.6 | 3 | 0.538 |
| RM1302 | 12 | 2.6 | 3 | 0.441 |
| RM7619 | 12 | 4.8 | 3 | 0.161 |
| RM101 | 12 | 8.8 | 3 | 0.669 |
| RM3331 | 12 | 23.5 | 4 | 0.678 |
| RM6947 | 12 | 24.0 | 2 | 0.467 |
| RM235 | 12 | 26.2 | 4 | 0.727 |
| RM313 | 12 | na | 2 | 0.632 |
| RM19 | 12 | na | 2 | 0.425 |
| RM8250 | na | na | 3 | 0.563 |
| RM1208 | na | na | 2 | 0.437 |

^1^Map position from Gramene Annotated Nipponbare Sequence 2009.

na = not available
