# Supplementary material for: Genetic variation in Southern USA rice genotypes for seedling salinity tolerance
Source: Front Plant Sci. 2015 May 27;6:374. doi: 10.3389/fpls.2015.00374 (PMC4444739; doi:10.3389/fpls.2015.00374)
Supplement: Supplementary file 3 [file Table3.DOCX]

Suppl. Table S3 Posterior probability of membership in salinity groupings by linear dicriminant analysis.

| Genotype | From Salinity Group^$^ | Classified into Salinity Group | HS | HT | MT | S | T |
| --- | --- | --- | --- | --- | --- | --- | --- |
| Hasawi | HT | HT | 0 | 1 | 0 | 0 | 0 |
| Cherivieruppu | HT | HT | 0 | 0.9999 | 0 | 0 | 0.0001 |
| Pokkali | HT | HT | 0 | 0.9995 | 0.0001 | 0 | 0.0004 |
| NonaBokra | HT | HT | 0 | 0.9981 | 0.0009 | 0 | 0.0011 |
| FL478 | HT | HT | 0 | 0.9824 | 0.0004 | 0 | 0.0173 |
| FL378 | HT | HT | 0 | 1 | 0 | 0 | 0 |
| TCCP266 | HT | HT | 0 | 0.7921 | 0.0025 | 0 | 0.2054 |
| IRRI147 | HT | HT | 0.0002 | 0.9968 | 0.0020 | 0 | 0.0011 |
| Ketumbar | HT | HT | 0 | 0.9977 | 0.0022 | 0 | 0.0001 |
| Damodar | HT | HT | 0 | 1 | 0 | 0 | 0 |
| Getu | MT | MT | 0.0002 | 0.0608 | 0.6837 | 0 | 0.2554 |
| CSR | T | T | 0 | 0.1753 | 0.0031 | 0 | 0.8216 |
| PSBRC50 | HT | HT | 0 | 0.9900 | 0.0025 | 0 | 0.0075 |
| IR1702 | MT | T^#^ | 0.0123 | 0.2863 | 0.2720 | 0.0002 | 0.4293 |
| IR944 | T | T | 0 | 0.0028 | 0.0364 | 0 | 0.9607 |
| IR2706 | S | S | 0.0042 | 0 | 0 | 0.9958 | 0 |
| Nipponbare | T | MT^#^ | 0.0113 | 0.0011 | 0.5314 | 0 | 0.4562 |
| Geumgangbyeo | T | T | 0 | 0.0045 | 0.0089 | 0 | 0.9865 |
| IR29 | HS | HS | 0.7660 | 0 | 0.0079 | 0.2259 | 0.0001 |
| Cocodrie | S | S | 0.0167 | 0 | 0 | 0.9833 | 0 |
| R609(MG) | T | T | 0.0033 | 0 | 0.1668 | 0.0001 | 0.8299 |
| LAH10 | T | T | 0.0011 | 0.0001 | 0.0300 | 0 | 0.9688 |
| LA0802140 | HS | HS | 0.7771 | 0 | 0.0117 | 0.2112 | 0 |
| Cheniere | T | T | 0.0108 | 0 | 0.0562 | 0 | 0.9330 |
| Bengal | S | S | 0.0201 | 0 | 0 | 0.9799 | 0 |
| CL152 | HS | HS | 0.6536 | 0 | 0.1040 | 0.2407 | 0.0017 |
| RoyJ | S | S | 0.2727 | 0 | 0.0519 | 0.6317 | 0.0437 |
| Rey | HS | HS | 0.8067 | 0 | 0.1787 | 0.0074 | 0.0071 |
| CL142 | S | S | 0.1774 | 0 | 0.0001 | 0.8226 | 0 |
| Mermentau | S | S | 0.0016 | 0 | 0 | 0.9984 | 0 |
| Jupiter | MT | MT | 0.0576 | 0 | 0.9388 | 0.0002 | 0.0034 |
| Wells | HS | HS | 0.8667 | 0 | 0.0163 | 0.1169 | 0.0001 |
| Catahoula | HS | HS | 0.8801 | 0 | 0.0548 | 0.0650 | 0.0001 |
| CL151 | S | S | 0.2056 | 0 | 0.0002 | 0.7942 | 0 |
| Jazzman | MT | MT | 0.1295 | 0 | 0.8686 | 0.0002 | 0.0016 |
| Neptune | MT | MT | 0.1843 | 0 | 0.8049 | 0.0007 | 0.0101 |
| Caffey | MT | MT | 0.2197 | 0.0001 | 0.6778 | 0.0018 | 0.1006 |
| Templeton | MT | MT | 0.1232 | 0.0001 | 0.8427 | 0.0002 | 0.0338 |
| Taggert | S | S | 0.0283 | 0 | 0.0015 | 0.9702 | 0 |
| Jazzman2 | S | HS^#^ | 0.9617 | 0 | 0.0050 | 0.0333 | 0 |
| Jes | HS | HS | 0.9005 | 0 | 0.0175 | 0.0819 | 0 |
| CL162 | MT | MT | 0.0140 | 0.0075 | 0.9468 | 0 | 0.0317 |
| CL181 | HS | HS | 0.9484 | 0 | 0.0237 | 0.0279 | 0 |
| CL111 | HS | HS | 0.9203 | 0 | 0.0733 | 0.0063 | 0.0001 |
| CL131 | HS | HS | 0.8603 | 0 | 0.1042 | 0.0333 | 0.0022 |
| Cypress | MT | MT | 0.0242 | 0 | 0.8615 | 0 | 0.1143 |
| CL161 | HS | HS | 0.9659 | 0 | 0.0288 | 0.0053 | 0 |
| LA0702085 | S | S | 0.0013 | 0 | 0 | 0.9987 | 0 |
| CL261 | S | S | 0.1518 | 0 | 0 | 0.8482 | 0 |

^#^ Rice genotypes misclassified

^$^ HT, Highly tolerant; T, Tolerant; MT, Moderately tolerant; S, Susceptible; HS, Highly susceptible
