# Supplementary material for: Genetic variation in Southern USA rice genotypes for seedling salinity tolerance
Source: Front Plant Sci. 2015 May 27;6:374. doi: 10.3389/fpls.2015.00374 (PMC4444739; doi:10.3389/fpls.2015.00374)
Supplement: Supplementary file 4 [file Table4.DOCX]

Suppl. Table S4 Canonical discriminant analysis between salinity grouping and trait responses to salinity stress.

| Canonical Discriminant Function | Canonical Correlation | Squared Canonical Correlation | Proportion of variance explained | Test of Significance |
| --- | --- | --- | --- | --- |
|  |  |  |  | Pr > F |
| 1 | 0.943 | 0.889 | 0.811 | <.0001 |
| 2 | 0.742 | 0.551 | 0.124 | <.0001 |
| 3 | 0.579 | 0.336 | 0.051 | 0.0035 |
| 4 | 0.349 | 0.122 | 0.014 | 0.1371 |
