# Supplementary material for: Genetic variation in Southern USA rice genotypes for seedling salinity tolerance
Source: Front Plant Sci. 2015 May 27;6:374. doi: 10.3389/fpls.2015.00374 (PMC4444739; doi:10.3389/fpls.2015.00374)
Supplement: Supplementary file 5 [file Table5.DOCX]

Suppl. Table S5 Total canonical structure of canonical discriminant function and class means of salinity group to canonical discriminant function.

| Variable^¥^ | Can1 | Can2 |
| --- | --- | --- |
| SIS | 0.87 | 0.10 |
| Ch_R | 0.82 | 0.49 |
| ShL_R | 0.59 | -0.49 |
| Ion_leak | 0.66 | -0.40 |
| Sh_K | -0.73 | 0.48 |
| Sh_Na/K | 0.77 | -0.21 |
|  |  |  |
| Salinity Group^$^ |  |  |
| HS | 1.86 | -0.23 |
| HT | -3.96 | 1.17 |
| MT | -0.34 | -0.81 |
| S | 3.37 | 0.90 |
| T | -1.55 | -1.84 |

^¥^ SIS= salt injury score; Chl_R= % reduction in chlorophyll; ShL_R= shoot length % reduction; Ion_leak=index of injury by ion leakage; Sht_K= shoot potassium content; Sh_Na/K= Na/K ratio in shoot.

^$^ HT, Highly tolerant; T, Tolerant; MT, Moderately tolerant; S, Susceptible; HS, Highly susceptible
