# Supplementary material for: Genetic variation in Southern USA rice genotypes for seedling salinity tolerance
Source: Front Plant Sci. 2015 May 27;6:374. doi: 10.3389/fpls.2015.00374 (PMC4444739; doi:10.3389/fpls.2015.00374)
Supplement: Supplementary file 6 [file Table6.DOCX]

Suppl. Table S6 P-value for pairwise comparison of LS means between salinity groups (i/j).

| i | j | SIS | Chl_R | ShL_R | Ion_leak | Sh_K | Sh_Na/K | Overall pair contrast |
| --- | --- | --- | --- | --- | --- | --- | --- | --- |
| HT | T | 0.868 | 0.436 | 0.012 | <.0001 | <.0001 | 0.072 | <.0001 |
| HT | MT | 0.002 | 0.244 | <.0001 | 0.016 | 0.000 | 0.000 | <.0001 |
| HT | S | <.0001 | <.0001 | 0.000 | <.0001 | <.0001 | <.0001 | <.0001 |
| HT | HS | <.0001 | <.0001 | <.0001 | 0.000 | <.0001 | <.0001 | <.0001 |
| T | MT | 0.087 | 0.009 | 0.694 | 0.144 | 0.949 | 0.461 | 0.007 |
| MT | S | 0.002 | <.0001 | 0.948 | 0.023 | 0.462 | 0.589 | <.0001 |
| S | T | <.0001 | <.0001 | 0.961 | 0.989 | 0.931 | 0.026 | <.0001 |
| HS | S | 1.000 | 0.015 | 0.919 | 0.339 | 0.999 | 0.992 | 0.001 |
| HS | MT | 0.002 | 0.061 | 1.000 | 0.649 | 0.319 | 0.829 | 0.001 |
| HS | T | <.0001 | <.0001 | 0.626 | 0.756 | 0.839 | 0.067 | <.0001 |

SIS= salt injury score; Chl_R= % reduction in chlorophyll; ShL_R= shoot length % reduction; Ion_leak=index of injury by ion leakage; Sht_K= shoot potassium content; Sh_Na/K= Na/K ratio in shoot.
